# The 8.2 ka cooling event in coastal East Asia: High-resolution pollen evidence from southwestern Korea

**DOI:** 10.1038/s41598-018-31002-7

**Published:** 2018-08-20

**Authors:** Jungjae Park, Jinheum Park, Sangheon Yi, Jin Cheul Kim, Eunmi Lee, Qiuhong Jin

**Affiliations:** 1 Department of Geography, Seoul National University, Sillim-dong, Gwanak-gu, Seoul, 151-742 Republic of Korea; 2 Institute for Korean Regional Studies, Seoul National University, Sillim-dong, Gwanak-gu, Seoul, 151-742 Republic of Korea; 3 Geo-Environmental Hazards & Quaternary Geology Research Center, Korea Institute of Geoscience and Mineral Resources, Daejon, 305-350 Republic of Korea

## Abstract

In this study, we present a high-resolution multi-proxy record (pollen, magnetic susceptibility, and mean grain size) from Bigeum Island, South Korea, which mainly showed early Holocene paleoenvironmental change. Bigeum pollen records indicated that early Holocene climate variations in coastal East Asia were principally controlled by the Atlantic meridional overturning circulation. Most importantly, the 8.2 ka cooling event was clearly recognized for the first time in coastal East Asia, where few high-resolution proxy data, such as ice core and stalagmite δ^18^O records, are available. The insular vegetation in the study site was extremely susceptible to even short-term climate changes, such as the 8.2 ka cooling event, which allowed a detailed climate reconstruction from pollen data. Early Holocene climate teleconnections between coastal East Asia and other regions were identified through regional comparisons of Greenland, China, Brazil, Spain, Madagascar, and Korea. Coastal East Asia is one of world’s most populated regions and will be particularly vulnerable to future climate change. Accurate and detailed paleoclimate proxy data, such as the Bigeum pollen record, will therefore be increasingly important in this region.

## Introduction

Abrupt short-term Holocene climate change has been extensively studied by paleoclimatologists because of concerns that a similar sudden change might occur in the future^1,2^. There have been numerous papers published regarding the causes and consequences of the 8.2 ka cooling event^3–9^. Similar research is still actively underway^10–12^. The 8.2 ka event is suggested to have been triggered by a large increase of meltwater discharge from the glacial Lake Agassiz into the North Atlantic^3^. Although the event lasted for only ~150 years, it caused a substantial climate deterioration in the North Atlantic region. Future global warming is highly likely to accelerate the freshwater supply to the Arctic Ocean^13,14^. Proxy data for the 8.2 ka would enhance our ability to predict possible responses of the climate system to the melting of Arctic sea ice in the future^3^.

In East Asia, the 8.2 ka event is clearly reflected in the cave stalagmite δ^18^O data from central China, which shows a strong linkage between the Asian monsoon and North Atlantic climate^7,15,16^. However, these data may not be suitable for examining Holocene climate variations in coastal East Asia (i.e., eastern China, Taiwan, Japan, and Korea). Recent studies have shown that there is a considerable difference between late Holocene climate changes in inland East Asia and coastal East Asia^17–19^. According to these studies, coastal East Asia was more greatly influenced by variations in tropical Pacific sea surface temperatures (SSTs) than by the North Atlantic climate change.

Here we present a high-resolution multi-proxy record (pollen, magnetic susceptibility [MS], and mean grain size) from Bigeum Island, South Korea, which mainly shows the early Holocene paleoenvironmental change. The primary aim of this study was to determine if the 8.2 ka event occurred over coastal East Asia. Our pollen data were therefore compared with high-resolution paleoclimate proxy records from other regions.

## Study Area

**Figure 1:**
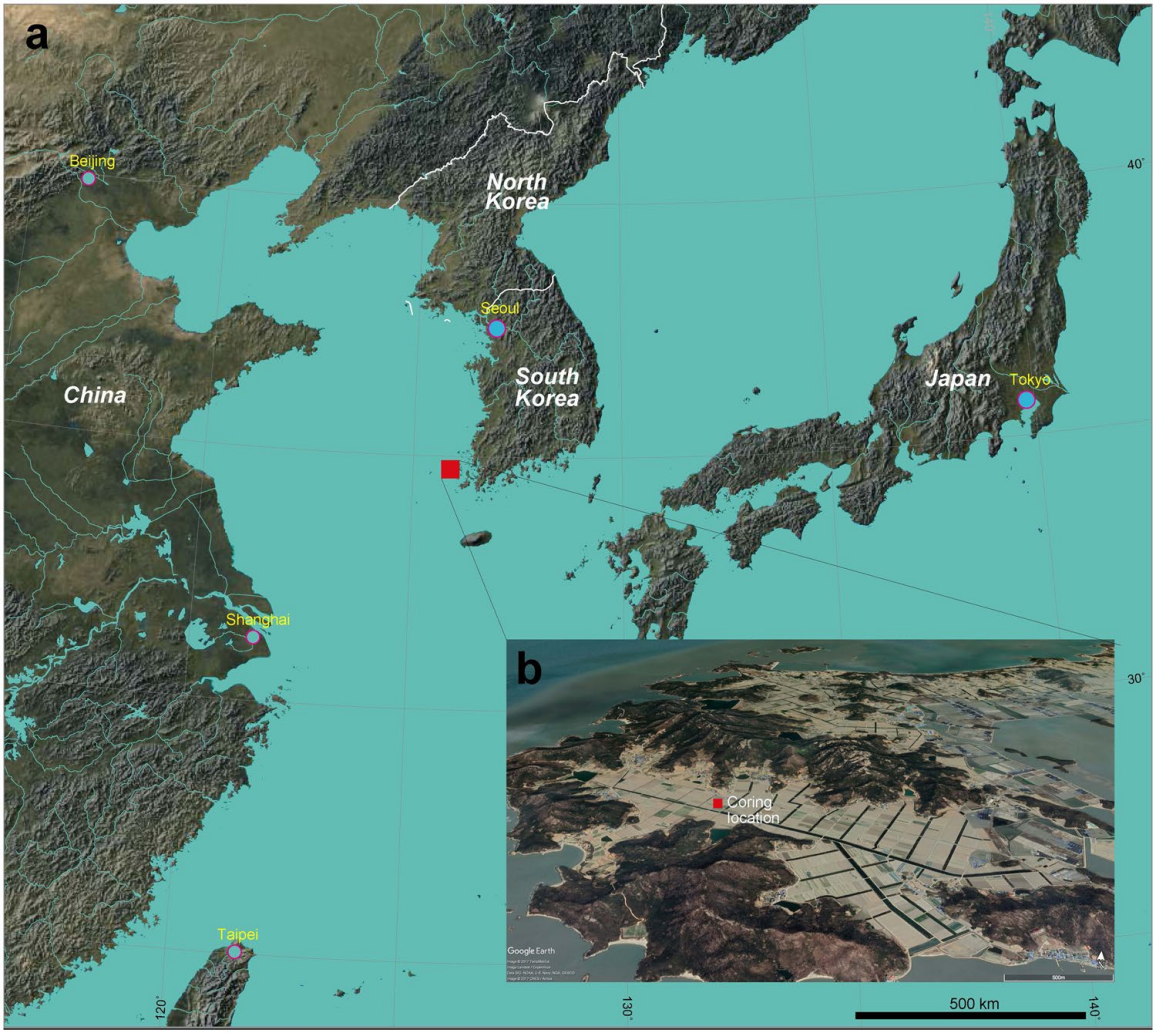
(**a**) Coastal region of East Asia and location of the study site (Bigeum Island). This map was modified from the UNAVCO Jules Verne Voyager (jules.unavco.org; UNAVCO Inc.) and is based on Generic Mapping Tools (GMT-5; gmt.soest.hawaii.edu). (**b**) The coring location in Bigeum Island. The bird’s-eye view image was generated using Google Earth (www.google.co.kr/intl/ko/earth/).

Bigeum Island, Sinan-Gun, Jeollanam-Do is located off the southwestern tip of the Korean Peninsula (Fig. 1). Dadohaehaesang National Park is located on the island and also extends across coastal areas of the South and West Sea of Korea. It is about 48 km^2^ in size, with a shoreline circumference and a long axis reaching ~88 and ~12 km, respectively^20^. Docho Island, the nearest inhabited island, is connected to Bigeum Island by a sea bridge.

On the west side of the island, strong wave energy has created beaches and sand dunes, along with sea cliffs. In contrast, the eastern part of the island consists of tidal flats, which have mostly been reclaimed since the 16^th^ century and used as rice paddies or salt ponds^21^.

A monsoon climate with hot/wet summers and cold/dry winters dominates in the Korean Peninsula. However, Bigeum Island is strongly influenced by a mild oceanic climate, and therefore the difference in the annual temperature range is not as large as in inland areas of Korea. The nearest weather station is on Heuksan Island located ~40 km west of the study site. Its monthly mean temperatures in January and August over the last 30 years were 3.3 and 24.5 °C, respectively, while the annul mean precipitation was 1,107 mm^22^. In addition, automatic weather station (AWS) data from the study site indicates that the annual mean temperature and precipitation were 14.6 °C and 1,134 mm from 2001 to 2010, respectively^23^.

People have used wood as fuel for salt pots since the Joseon Dynasty, with the result that vegetation around the salt farms has been largely destroyed^24^. Some forest has recovered since solar salt production began in the early 1900s; however, growth has been poor because of the strong sea breeze and barren soil. The vegetation is dominated by *Pinus thunergii* and *Alnus firma*. The latter was planted to prevent landslides. On Geumsung mountain (211 m), on the neighboring Docho Island, the flora mostly consists of *Pinus thunbergii*, *Eurya japonica*, and *Mallotus Japonicus* and weedy species such as *Imperata cylindrica*, *Smilax china*, *Hedera rhombea*, and *Dioscorea tokoro*^24^.

Freshwater wetland plants growing on Bigeum Island include Miscanthus sacchariflorus, Phragmites communis, Typha angustifolia, Leersia japonica, Paspalum distichum, Persicaria thunbergii, Hydrocharis asiatica, Nelumbo nucifera, Euryale ferox, Trapa japonica, Nymphaea tetragon, Spirodela polyrhiza, and Myriophyllum verticillatum^25^. According to a recent report from Jangsan Island ~20 km southeast of the study area, coastal wetlands are dominated by Phragmites communis, Zoysia sinica, Carex scabrifolia, Suaeda maritima, Suaeda asparagoides, Calystegia soldanella, and Vitex rotundifolia^26^.

## Results and Discussion

### Sediment chronology and sea level rise

**Figure 2:**
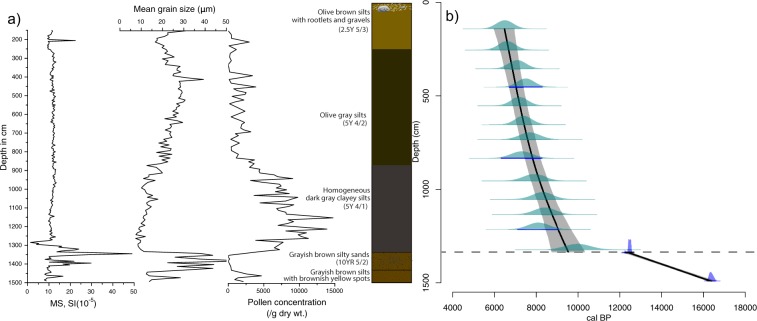
(**a**) Bigeum multi-proxy data (magnetic susceptibility (MS), mean grain size, pollen concentration) and the sediment stratigraphy (SI Text). (**b**) Fifteen dates from the Bigeum sediment core. A best age-depth model (black line), with a 95% confidence interval (gray shading) was established from 10 optically stimulated luminescence (OSL) dates and 2 radiocarbon dates, based on a smooth spline function fitted using Clam 2.2^44^. Three unused OSL dates are indicated by blue underlines. Age probability distributions are plotted in light green for OSL dates and in light blue for radiocarbon dates. Note the presence of an erosional conformity at 13.4 m.

No terrestrial plant materials were found throughout the core, and therefore we had no choice but to date bulk sediment samples. Thirteen samples were submitted to the Korea Institute of Geoscience and Mineral Resources (KIGAM) for OSL dating and two samples to Beta Analytic Inc. for AMS dating (Table 1 and Fig. 2b). However, three OSL dates that deviated from the overall trend (depths of 4.53, 8.34, and 12.15 m) were not used for establishment of the age-depth profile.

**Table 1:** OSL and Radiocarbon dates for Biguemdo sediments.

OSL Sample depth(cm)	Dose rate (Gy/ka)	Water content (%)	Equivalent dose (Gy)	Age (ka, two σ age)
142	3.30 ± 0.19	0.264	21.40 ± 0.10	6.5 ± 0.4
255	3.36 ± 0.19	0.303	22.24 ± 0.33	6.6 ± 0.4
355	3.23 ± 0.18	0.287	22.82 ± 0.12	7.1 ± 0.4
453	3.25 ± 0.18	0.293	24.38 ± 0.35	7.5 ± 0.4
553	3.34 ± 0.19	0.271	24.03 ± 0.36	7.2 ± 0.4
654	3.31 ± 0.19	0.291	24.32 ± 0.34	7.4 ± 0.4
734	3.28 ± 0.19	0.292	25.34 ± 0.45	7.7 ± 0.5
834	3.35 ± 0.20	0.273	24.42 ± 0.41	7.3 ± 0.5
955	3.18 ± 0.19	0.370	25.18 ± 0.29	7.9 ± 0.5
1054	3.19 ± 0.19	0.408	26.45 ± 0.62	8.3 ± 0.5
1135	3.11 ± 0.18	0.383	26.01 ± 0.31	8.4 ± 0.5
1215	3.19 ± 0.19	0.441	25.84 ± 0.46	8.1 ± 0.5
1323	3.35 ± 0.20	0.348	33.54 ± 0.75	10.0 ± 0.6
**AMS Sample depth(cm)**	**Material dated**	**Lab no.**	**Age (** ^**14**^ **C yr BP)**	**Two σ age range (cal yr BP)**
1340	Bulk sediments	BETA-471023	10500 ± 40	12590–12240
1490	Bulk sediments	BETA-471022	13590 ± 40	16580–16190

The most common methods for dating bulk sediments are radiocarbon and OSL dating. AMS data have limitations for estimating the age of coastal marine sediments because local and temporal differences in the marine reservoir effect need to be considered. The reservoir effect around the Korean Peninsula is not fully understood due to a lack of relevant studies^27^. OSL dates are free from the effects of old carbon, but have large 2σ age ranges, which can span several hundred years. Therefore, it is relatively difficult to ensure accuracy when assigning ages to the depth intervals of marine sediments. In this study, high-sampling resolution OSL dating (rather than radiocarbon dating) was conducted to minimize chronological problems, such as large deviation ranges and the effect of the marine radiocarbon reservoir.

According to the radiocarbon data, the terrestrial sediments in the bottom part of the core were deposited between ~16,500 and ~12,500 cal yr BP (hereafter BP). However, OSL data indicated that marine sediments began to be deposited at ~9,500 BP. It is highly likely that some of the upper part of the terrestrial sediment was eroded during the marine transgression. A slow and steady sedimentation, with negligible erosion, then occurred under the influence of relatively high sea level stands in the study site. The deceleration of sea level rise then caused rapid sedimentation from ~8,000 BP (SI Text). The Bigeum age model and core stratigraphy allowed us to hypothesize that sea levels rose rapidly between 9,500–8,000 BP, and slowly between 8,000–6,500 BP; they then stabilized from 6,500 BP, leading to little sedimentation at the study site.

### Pollen data

**Figure 3:**
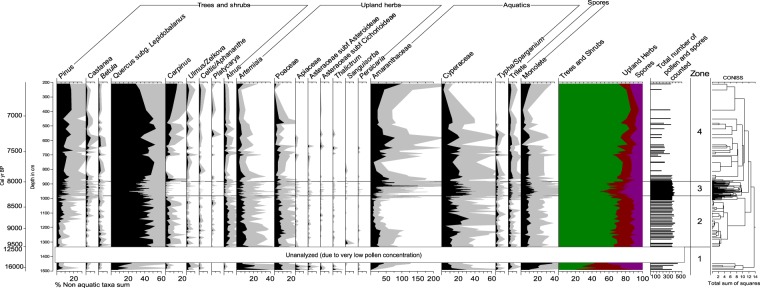
Selected pollen and spore taxa from Bigeum sediments. All percentages are based on the sum of all non-aquatic taxa.

In general, Bigeum pollen assemblages were dominated by *Quercus* and two coastal marsh taxa (Amaranthaceae and Cyperaceae). There was a high percentage content of Amaranthaceae in the pollen profile. Pollen records are described and discussed for each pollen zone as follows (Fig. 3):

#### Zone 1 (16,500–12,500 BP)

In this zone, statistically sufficient pollen grains were counted only in the early part (16,500–15,500 BP), and it was therefore difficult to derive meaningful information from the pollen assemblages. This period was mainly dominated by *Artemisia*, Poaceae, Amaranthaceae, Cyperaceae, and monolete fern spores. Arboreal taxa such as *Quercus* were found in insignificant quantities, indicating that the East Asian summer monsoon (EASM) remained weak during the last deglaciation.

#### Zone 2 (9,500–8,300 BP)

As mentioned earlier, the sediment deposited between 12,500–9,500 BP was presumably eroded by the marine transgression around ~9,500 BP. This sea level rise, often referred to as MWP 1c, is believed to have been caused by the discharge from Lake Agassiz at the margin of the Laurentide Ice Sheet^28,29^. In this zone, *Quercus* was predominant (>50%).

The percentage content of *Carpinus* and *Alnus* increased markedly from the previous zone, while *Artemisia* rapidly declined. In general, there was an opposite trend in the percentage change between the two coastal marsh taxa. Amaranthaceae displayed a steady decrease over almost the entire zone, while Cyperaceae increased. However, near the end of the zone, there was a sudden increase in Amaranthaceae, with a corresponding decline in Cyperaceae. The spores (trilete and monolete) were present at low frequencies, with little change occurring throughout the zone.

The pronounced increase in *Quercus*, *Carpinus*, and *Alnus*, and a decrease in herbs such as *Artemisia*, indicated that climatic conditions became warmer and wetter during the early Holocene. Changes in Amaranthaceae and Cyperaceae seem to have been associated with the sea level rise rather than climate change. The high percentage content of Amaranthaceae during the early part of this zone may have been attributable to the continuously high rate of sea level rise. The increasing influence of the sea would have been an advantage for highly salt-tolerant Amaranthaceae species, such as *Suaeda japonica* and *S*. *maritima*. A decline in Amaranthaceae and an increase in Cyperaceae during the middle of the zone indicated that the rate of sea level rise was slowing down.

The rapid rise in Amaranthaceae from ~8,400 BP indicated that there was another massive sea level rise. The Lake Agassiz discharge was suggested to have consisted of two episodes^30^. The earlier freshwater outburst at ~8,400 BP may have led to an approximately one-meter rise in sea level along the coast of East Asia, according to numerical model studies^31^. The later flood is believed to have been the main cause of the 8.2 ka cooling event^29,30,32^.

#### Zone 3 (8,300–8,000 BP)

Zone 3 was characterized by a rapid decline in *Quercus*. There was also a similar decrease in *Carpinus* and *Alnus*. In contrast, monolete spores and Cyperaceae increased in frequency. Herbs such as Apiaceae, *Sanguisorba*, and *Persicaria* also slightly increased compared to the previous zone, although not by as much as monolete spores. The latter part of the zone is of interest because Amaranthaceae, which began to rise in frequency at ~8,400 BP near the end of zone 2, increased tremendously, to more than 150% of the sum of non-aquatic taxa.

Zone 3 included the 8.2 ka cooling event, which resulted in a cold and/or dry climate throughout the northern hemisphere (NH). The rapid decline in *Quercus*, and the increase in spores and herbs, were indicative of a sudden shift in the East Asian monsoon (EAM). Climate deterioration presumably reduced the density of trees, and the newly exposed land surface was likely to have been colonized by ferns or herbaceous plants. In addition, the marked rise in Amaranthaceae was likely due to the sea level rise, which was presumably triggered by the aforementioned meltwater pulse (MWP) at ~8,400 BP.

The variations in Cyperaceae frequency seem to have been mostly related to sea level changes. However, it cannot be ruled out that the Cyperaceae frequency also reflects climate change, given its significant increase at ~8,200 BP. Sedge populations may have expanded on vegetated coastal dunes at the expense of trees. However, Cyperaceae is usually not an appropriate pollen indicator of climate change because it is largely indicative of local environmental changes.

#### Zone 4 (8,000–6,500 BP)

High sedimentation rates and consequent low pollen concentrations did not permit a sufficiently detailed analysis of this zone, placing limits on paleoclimate reconstruction. The important characteristic of this zone was the dominance of *Quercus*. Other arboreal taxa, such as *Pinus* and *Carpinus*, also increased appreciably toward the latter part of the zone. Conversely, there was a steady decline in the abundance of herbs and spores. The decrease in *Artemisia* was particularly evident and many herb taxa disappeared in the latter part. There was a gradual decrease in Cyperaceae, whereas Amaranthaceae increased between 7,700 BP and 7,200 BP, and after ~6,700 BP.

The gradual increase in tree taxa and decrease in herbs and spores indicated that climatic conditions were continuously ameliorated by strengthening of the EASM. This zone is likely to have belonged to the Holocene Climate Optimum in the Korean Peninsula^33,34^. In addition, the increase in Amaranthaceae between 7,700–7,200 BP might have been related to another MWP (MWP 1d)^28,29,35^.

### The 8.2 ka cooling event

Bigeum pollen records demonstrated distinct vegetation changes around 8,200 BP. A rapid drop in arboreal pollen frequency and a corresponding increase in fern spores indicated that the abrupt dry and/or cold event significantly disturbed the vegetation in the study area. In particular, enhanced aridity was clearly indicated by the decline in *Alnus*, which commonly colonizes water-logged soil and thus sensitively responds to shifts in the water table. The 8.2 ka cooling event had a noticeable impact on the Korean Peninsula.

**Figure 4:**
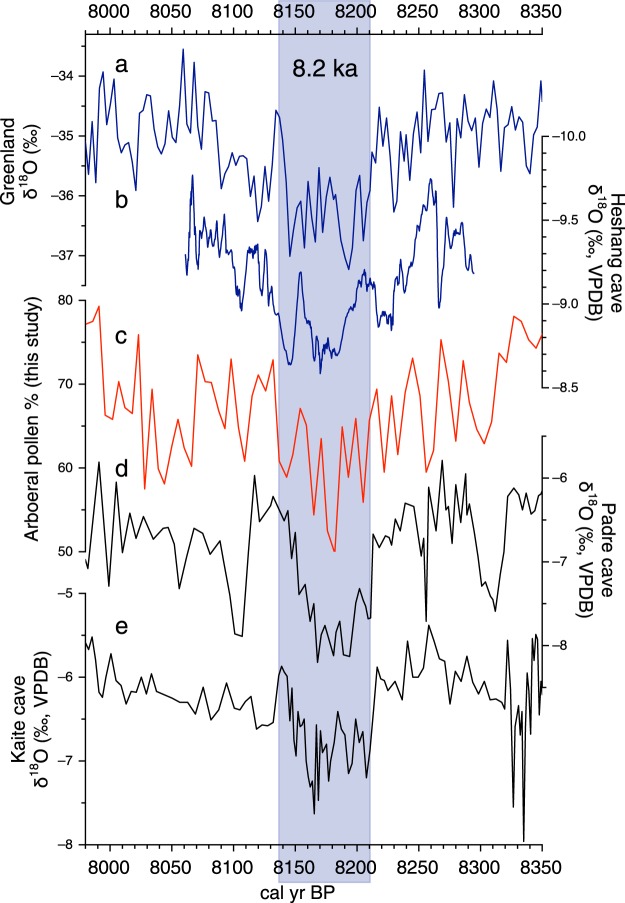
Comparison among Greenland ice core δ^18^O records^6^ (**a**), stalagmite δ^18^O records from Heshang cave, central China^7^ (**b**), the percentage content of total arboreal pollen from Bigeum Island (**c**), stalagmite δ^18^O records from Padre cave, eastern Brazil^7^ (**d**), and stalagmite δ^18^O records from Kaite Cave, Spain^8^ (**e**). The 8.2 ka cooling event is indicated by the transparent blue bar. This diagram is produced using pro Fit 7.0.7 software (www.quansoft.com).

Pollen assemblages of the 8.2 ka event are sufficiently detailed to enable a comparison with other important proxy data, and to examine the temporal structure of the cooling event (Figs 4 and S2). As expected, Bigeum pollen data displayed similarities to other global paleoclimate proxy data, including ice core δ^18^O records from Greenland^6^ (Fig. 4a) and cave stalagmite δ^18^O records from southern China^7^ (Fig. 4b), eastern Brazil^7^ (Fig. 4d), Spain^8^ (Fig. 4e), and northwestern Madagascar^12^ (Fig. S2c).

The 8.2 ka cooling event is characterized by its “double-plunge” structure, according to cave δ^18^O records from Greenland and China^7^. Our total arboreal pollen (AP) percentages indicate a possible teleconnection between the North Atlantic climate^6^ and the EAM (Fig. 4a–c). Interestingly, there was also strong similarity in the variations between total AP percentages and cave δ^18^O records from eastern Brazil^7^ and Madagascar^12^. As the inter tropical convergence zone (ITCZ) migrated southward during the 8.2 ka cooling event, the climate in the Korean Peninsula became drier. In contrast, relatively wetter conditions prevailed over equatorial Brazil and northwestern Madagascar in the southern hemisphere. Spain is also likely to have experienced relatively humid conditions during the cooling event, because moist westerlies were enhanced over the Iberian Peninsula due to the more equatorward position of the ITCZ.

Our pollen records provide reliable evidence for the occurrence of an 8.2 ka cooling event in the study area, given the strong links with other high-resolution paleoclimate data. They show a marked adjustment of the vegetation to both the abrupt drying and the subsequent wetting, with no discernible time delay. However, considering the dating uncertainties for Bigeum sediment, it is not feasible to be conclusive regarding how promptly vegetation responded to the 8.2 ka event in the study area. We admit that the identification of the 8.2 ka event in our records was partly based on “wiggle matching” as the OSL dates have considerable age uncertainties.

Large tree populations can usually tolerate a changing climate, unless its impact surpasses their physiological limits to adaptation^36^. However, it is likely that the insular tree flora in the study area responded quite sensitively to even brief climate changes, such as the 8.2 ka cooling event. Most of the island forests are fragile ecosystems that are highly vulnerable to disturbances. However, the marked decline in arboreal pollen at 8.2 ka cannot be fully explained by the low resilience of island forests. The drying and/or cooling over the study area may have been almost comparable to the climate shift in the North Atlantic. Although it was difficult to ascertain which explanation was more pertinent, it is clear that our pollen data, for the first time, provide a clear indication of the 8.2 ka cooling event occurring in coastal East Asia. There have been a few sediment proxy records suggesting the occurrence of the event in this region, but they were all ambiguous in terms of the event’s timing and structure^37–39^.

It is believed that, during the Holocene, the EAM was mainly modulated by the orbitally related decline in NH insolation, variations in solar output, and the Atlantic meridional overturning circulation^15,40^. However, recent studies suggest that mid-to late-Holocene climate change in coastal East Asia was principally controlled by the SST variability of the western tropical Pacific, unlike inland areas in East Asia^18,19^. The increase in El Niño–Southern Oscillation (ENSO) activity from ~5,500 BP^41,42^ could be attributed to the decline in NH insolation during the late Holocene^43^, and is likely to have been a major cause of the different climate mechanisms that operated in the two regions.

However, variations in the early Holocene EAM over the study area had a strong association with the climate change triggered in the North Atlantic, before the tropical Pacific Ocean exerted a significant modulatory effect on it. The 8.2 ka vegetation change shown in Bigeum pollen records clearly indicates that the oceanic Atlantic meridional overturning circulation was the main driver of early Holocene climate change in the study area.

## Conclusion

To determine the characteristics of short-term climate changes, such as the 8.2 ka cooling event, we need high-resolution proxy data with a reliable chronology. The most suitable records for such work would be δ^18^O data from stalagmites and ice cores, which are unfortunately not available in coastal East Asia. Sediment pollen data, which is a useful paleoclimate proxy, is relatively easy to obtain in the region. However, substantial effort and time are typically required for high-resolution pollen analysis. Climate reconstructions based on pollen-vegetation-climate relationships are not as accurate as direct reconstructions from ice cores or speleothem δ^18^O values. The time lag in the response of vegetation to climate change is another limitation when using fossil pollen as a paleoclimate proxy, especially when short-term climate variability is investigated.

Nevertheless, the brief cooling event at 8.2 ka is clearly exhibited in Bigeum pollen data. For the first time, we herein presented evidence of its occurrence in coastal East Asia, without the use of Holocene stalagmite and ice core samples. The insular vegetation in the study area is extremely susceptible to even short-term climate changes. Coastal East Asia, one of world’s most populated regions, will be particularly vulnerable to future climate change. To mitigate the likely harm to humans arising from natural hazards, climate change should be properly modelled. Detailed paleoclimate proxy data, such as our pollen records, will therefore be increasingly important for studies of this region.

## Materials and Methods

### Core materials and chronology

In May 2016, a 15-m-long sediment core in 1-m-long sections was taken from a reclaimed paddy field in Bigeum Island with a hydraulic corer (34°44′05″N, 125°54′36″E; Fig. 1). Two bulk sediment samples were submitted to Beta Analytic (Miami, FL, USA) for accelerator mass spectrometry (AMS) radiocarbon dating (Table 1). The calibrated age range was determined using Clam 2.2 software^44^ and the IntCal13 dataset^45^.

For optically stimulated luminescence (OSL) dating, 4–11 μm diameter quartz was extracted from 13 subsamples. Sodium pyrophosphate, HCl, and H_2_O_2_ were used to remove clays, carbonates, and organics, respectively. Then, we etched samples in H_2_SiF_6_ for 14 days to chemically remove feldspar. The OSL measurements were made using a TL-DA-20 reader (Risø DTU, Roskilde, Denmark) equipped with a blue light-emitting diode (470 ± 20 nm) stimulation source. Irradiation was provided by a ^90^Sr/^90^Y beta source delivering approximately 0.09 grays (Gy) s^−1^. A 9635 QA photomultiplier tube (Thorn EMI, Fairfield, NJ, USA) and a 7.5-mm-thick U-340 filter were used for photon detection. The radionuclide contents were measured using low-level high-resolution gamma spectrometry. Conversion to dose rates was based on the data reported by Olley *et al*.^46^. The water content (weight of water/weight of dry sediment) was used for dose rate correction. Cosmic ray contributions were calculated using the equations of Prescott and Hutton^47^. The single-aliquot regenerative-dose (SAR) procedure^48^ was applied to chemically purify quartz grains with diameters of 4–11 and 90–250 μm.

### Multiproxy data

A total of 127 samples were taken for pollen analysis at intervals of 2–10 cm. Pollen was extracted using standard palynological procedures (Faegri and Iversen, 1989). The samples were successively treated with HCL, KOH, HF, and acetolysis and sieved with 180 and 10 μm mesh filters to remove large organic debris and fine fractions after the KOH treatment. Pollen counts were obtained using a Leica microscope with a 40× objective at a total magnification of 400×. A minimum of 300 pollen grains were counted from each slide. However, 16 samples lacked sufficient pollen concentrations to permit counting to 300. In those samples, a minimum of 100, 150, or 200 pollen grains were counted.

A pollen diagram was produced using Tilia^49^. *Lycopodium* spore tablets were added to each sample to calculate pollen concentrations. The total sum of the non-aquatic pollen and spores was used as the basis to calculate all percentages shown in the pollen diagram. A stratigraphically constrained cluster analysis was also conducted based on the percentages of non-aquatic taxa using CONNIS, and four stratigraphic zones and two subzones were delineated.

The MS was measured at 5 cm intervals using an MS2 meter (Bartington Instruments, Witney, UK), and mean grain size was measured at 10 cm intervals using a Mastersizer 2000 laser diffraction particle size analyzer (Malvern Instruments, Malvern, UK). For the latter analysis, about 300 mg of sample was treated with 35% H_2_O_2_ to remove organic matter, and with 1 N HCl under a hot water bath to remove carbonates.

## Electronic supplementary material

Supplementary Information

## Data Availability

The datasets generated during and/or analysed during the current study are available from the corresponding author on reasonable request.
