# Supplementary material for: The 8.2 ka cooling event in coastal East Asia: High-resolution pollen evidence from southwestern Korea
Source: Sci Rep. 2018 Aug 20;8:12423. doi: 10.1038/s41598-018-31002-7 (PMC6102300; doi:10.1038/s41598-018-31002-7)
Supplement: Supplementary file 1 — Supplementary Information [file 41598_2018_31002_MOESM1_ESM.pdf]

Supplementary Information

The 8.2 ka cooling event in coastal East Asia: High-resolution pollen evidence from southwestern Korea

Jungjae Park [a,b,1], Jinheum Park [a], Sangheon Yi [c,2], Jin Cheul Kim [c], Eunmi Lee [c], Qiuhong Jin [a]

[a] Department of Geography, Seoul National University, Sillim-dong, Gwanak-gu, Seoul, 151-742, Republic of Korea

[b] Institute for Korean Regional Studies, Seoul National University, Sillim-dong, Gwanak-gu, Seoul, 151-742, Republic of Korea

[c] Geo-Environmental Hazards & Quaternary Geology Research Center, Korea Institute of Geoscience and Mineral Resources, Daejon, 305-350, Republic of Korea

[1] Corresponding author

Email address: jungjaep@snu.ac.kr

Telephone number: 82-2-880-6140

Fax number: 82-2-876-9498

[2] Corresponding author

Email address: shyi@kigam.re.kr

Telephone number: 82-10-3854-9522

SI Text

Sediment stratigraphy

The Bigeum Island sediment core was 15-m-long and could be divided into five sections depending on the physical characteristics of the sediments (Fig. 2a and Fig. S1). The first section between the depths of 15–14.3m was composed mostly of silts, while the second section (14.3–13.4m) consisted of sands and coarse silts. These two sections had a grayish brown color, which may have resulted from exposure to the air after deposition on natural levees or backswamps. In general, higher MS values are found in less weathered, and thus coarser, grained sediments. The increasing MS and mean grain size in the second section were indicative of an increased rate of erosion, attributable to enhanced precipitation in the Bølling-Allerød warm period. In this section, there were few pollen grains and a high percentage of sands (> 40%).

The third section (13.4–8.7m) consisted of oceanic fine sediments (fine silt and clay) having a dark gray color. There was a pronounced increase in pollen concentrations in the lower part of this section. The concentrations remained high throughout the section. There was no noticeable change in MS values. As sea levels rose during the early Holocene, the study site changed from a terrestrial to ocean environment. The amount of accommodation space was amplified by the rapid sea level rise in the sedimentary basin. As a result, sedimentation rates significantly declined, leading to increased pollen concentrations.

The fourth section (8.7–2.5m) was mainly composed of fine silts (20–30 mm) having an olive gray color. There was an increase in the mean grain size from a depth of 8.7 m, above which the rough surfaces of the sediment were clearly apparent on core

photographs (Fig. S1). The MS values were almost the same as in the previous section. The lack of change in the MS values from the previous section indicated that this section also consisted of ocean sediments. Pollen concentrations, which markedly decreased in the lower part of the section, remained very low throughout the section. Because the sedimentary accommodation space was reduced by the deceleration in sea level rise after ~ 8,000 cal yr BP, a consequent increase in sedimentation rates led to a decline in pollen concentrations. The increased mean grain sizes indicated that the sedimentation mostly occurred on tidal wetlands near creeks after the deceleration of sea level rise. The sediment color changed from dark gray to olive gray, which could be attributed to a relative lowering of the sea level. Above a depth of 2.5 m, there were disturbed sediments having an olive brown color; these arose from exposure to the air after sea levels began to stabilize at ~ 7,000 cal yr BP and were later reclaimed for agriculture.

SI Figures

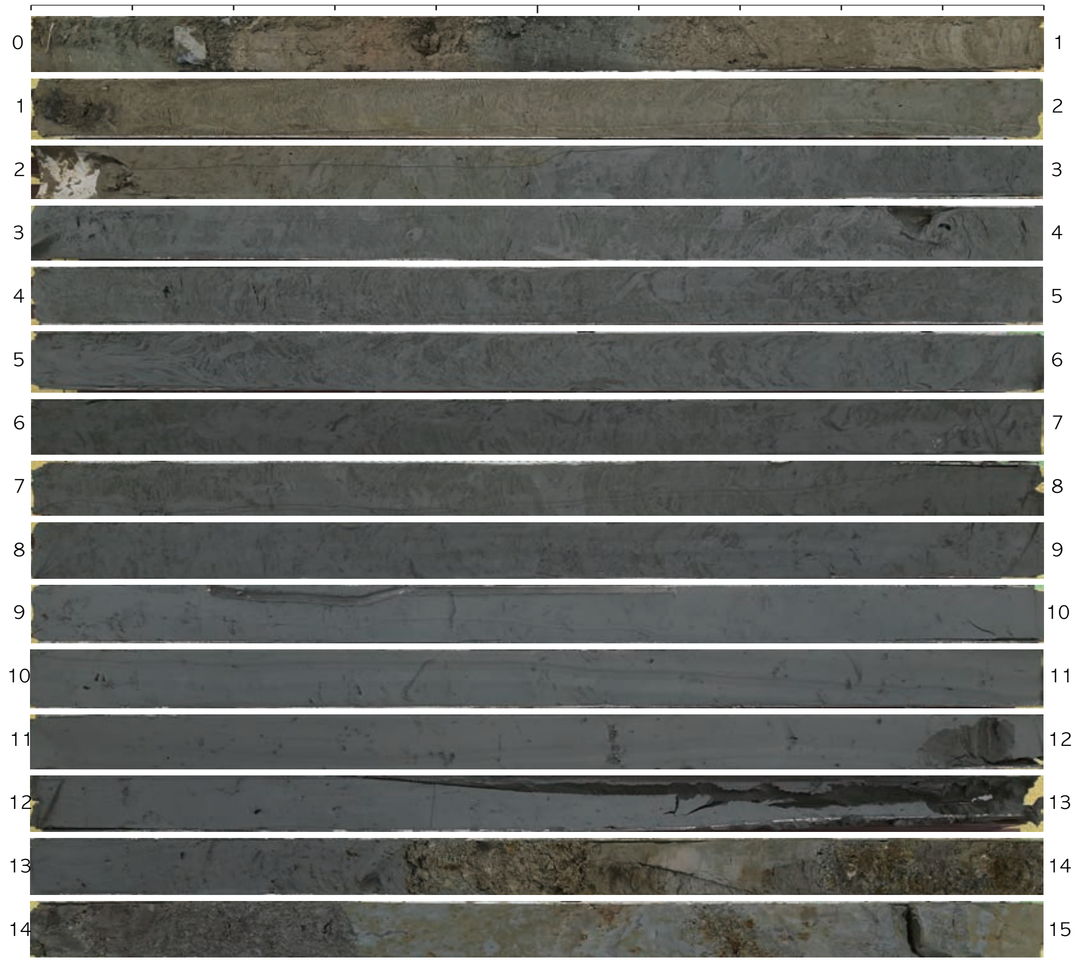

Fig. S1. A digital image of the 15 m-long Bigeum sediment core.

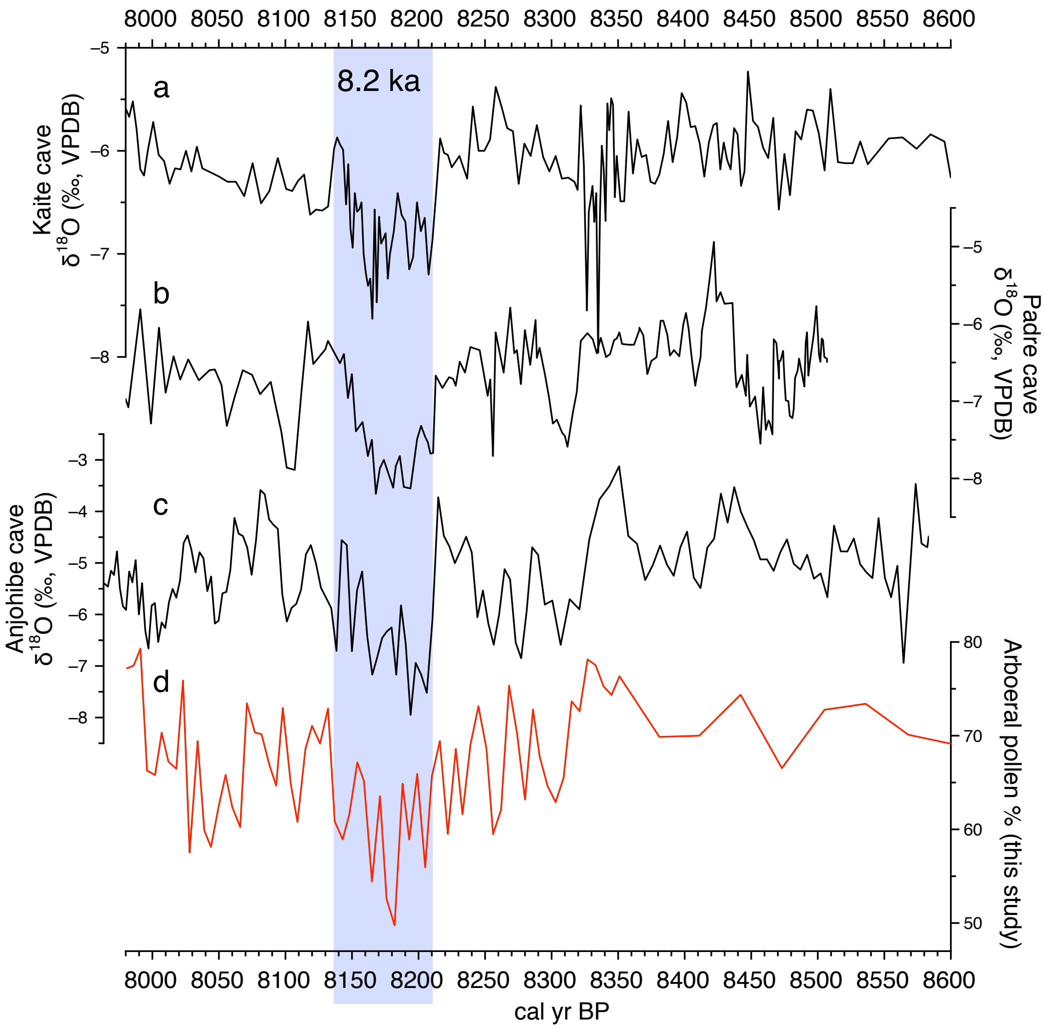

Fig. S2. Comparison among stalagmite $\delta^{18}O$ records from Kaite Cave, Spain [1] (a); stalagmite $\delta^{18}O$ records from Padre cave, eastern Brazil [2] (b); stalagmite $\delta^{18}O$ records from Anjohibe cave, northwestern Madagascar [3] (c); and the percentage content of total arboreal pollen from Bigeum Island (d). The 8.2 ka cooling event is indicated by the transparent blue bar. Note that temporal adjustments (~15 years) were applied to Anjohibe $\delta^{18}O$ records (c) to match a point associated with the beginning of the 8.2 ka event. Its close linkage with Biguem pollen data is particularly noticeable. This diagram is produced using pro Fit 7.0.7 software (www.quansoft.com).

References

1 Domínguez-Villar, D. *et al.* Oxygen isotope precipitation anomaly in the North Atlantic region during the 8.2 ka event. *Geology* **37**, 1095-1098 (2009).

2 Cheng, H. *et al.* Timing and structure of the 8.2 kyr BP event inferred from δ18O records of stalagmites from China, Oman, and Brazil. *Geology* **37**, 1007-1010 (2009).

3 Voarintsoa, N. R. G. *et al.* Three distinct Holocene intervals of stalagmite deposition and nondeposition revealed in NW Madagascar, and their paleoclimate implications. *Climate of the Past* **13**, 1771 (2017).
